# Patterns of Rheumatic Heart Disease and Treatment Practices at Tertiary Care Center in Nepal: A Descriptive Cross-sectional Study

**DOI:** 10.31729/jnma.5405

**Published:** 2020-10-31

**Authors:** Rajesh Nepal, Madhab Bista, Sahadeb Prasad Dhungana

**Affiliations:** 1 Department of Internal Medicine and Cardiology Unit, Nobel Medical College Teaching Hospital, Biratnagar, Nepal

**Keywords:** *rheumatic heart disease*, *spectrum analysis*, *treatment*

## Abstract

**Introduction:**

Rheumatic heart disease is a sequel of rheumatic fever which causes heart valve damage. This study was conducted to look at the pattern of valve lesions and treatment practices in patients with rheumatic heart disease.

**Methods:**

A cross-sectional study conducted at the tertiary care center with a diagnosis of rheumatic heart disease from July 2018 to January 2020 by convenient sampling. Ethical clearance was obtained from the Institutional Review Committee (ref no. 55/2018). Data were analyzed by using Statistical package for social sciences version 20.

**Results:**

Out of 600 patients, 428 (71.3%) were female. The mean age was 44.24±14.24 years. The isolated mitral valve was affected in 280 (46.6%). Dual involvement of mitral and aortic valve was present in 294 (49%). Only 14 (2.3%) had involvement of isolated aortic valve involvement. Overall, mitral stenosis was the most common abnormality 508 (84.6%) followed by mitral regurgitation 418 (69.6%), aortic regurgitation 320 (53.3%), and aortic stenosis 63 (10.5%). Assessment of the severity of lesions showed that 247 (41.2%) patients had severe mitral stenosis, 119 (19.8%) severe mitral regurgitation, 14 (2.3%) severe aortic stenosis, and 11 (1.8%) severe aortic regurgitation. Majority 493 (82.2%) were treated with medical therapies. Surgical procedures were performed in 51 (8.5%). The use of anticoagulation was in 212 (35.3%) of eligible patients.

**Conclusions:**

Mitral valve was affected commonly both in isolation and combination. The majority of patients who were eligible for cardiac interventions were treated medically with suboptimal use of anticoagulation and secondary prophylaxis.

## INTRODUCTION

Rheumatic heart disease (RHD) is an important preventable cause of cardiovascular disability and mortality in developing countries. According to world health organization (WHO), at least 15.6 million people worldwide have RHD.^1^ Of the 5,00,000 individuals who acquire acute rheumatic fever (ARF) every year, 3,00,000 go on to develop RHD and 233,000 deaths annually are attributed to ARF or RHD.^1^

Various studies have been published on the prevalence of RHD in Nepal in various journals.^2–5^ All these studies showed the prevalence of RHD among school children to be between 0.9-1.35 per thousand in different parts of Nepal. Single or combined valvular lesions are present with variable clinical presentations in RHD patients. There are limited data on the clinical spectrum of RHD and treatment practices from our population.

Therefore, this study was conducted to look at the pattern of valvular lesions and to find out treatment practices in RHD patients at tertiary care hospitals in the eastern part of Nepal.

## METHODS

This is a descriptive cross-sectional study conducted at Nobel Medical College Teaching Hospital who were at an outpatient or admitted at the cardiology unit from July 2018 to January 2020 after getting approval from the Institutional review committee (ref no. 55/2018). Six hundred patients (more than calculated sample size of 338) of RHD with age ≥12 years were included in the study. Patients with degenerative valvular disease and children with acute rheumatic fever below 12 years of age were excluded from the study. All the participants had signed the consent for the study. The diagnosis of RHD was made based on clinical history, examination, and echocardiogram. Electrocardiography and echocardiography were performed in each individual.

The minimum sample size of 338 was calculated based on the 33% hospital prevalence of RHD (around one-third of total admission in cardiology unit - educated guess).

$$\begin{array}{ll} \text{n} & \text{= }{\text{Z}}^{\text{2}}\times \text{p}\times \text{q}/{\text{e}}^{\text{2}}\\ & \text{= }{\left(1.96\right)}^{\text{2}}\times 0.33\times 0.67/{\left(0.05\right)}^{\text{2}}\\ & \text{= }3.84\times 0.22/0.0025\\ & =338 \end{array}$$

where,

n = required sample size
Z = 1.96 at 95% confidence interval
p = hospital prevalence of cardiac unit (33%-educated guess)
q = 1-p
e = margin of error, 5%

Data were collected with the inclusion of pre-specified clinical parameters in Performa by convenient sampling method. The data were collected and entered in MS-Excel 2007 and analyzed using the Statistical package for social sciences (SPSS) version 20 software. For descriptive statistics percentage, mean and standard deviation were calculated. The graphical and tabular presentation was made for appropriate variables.

All RHD patients underwent mode (M), 2-dimensional (2D), color flow and pulsed wave Doppler transthoracic echocardiography by Siemens echo machine. Echocardiograms were obtained at rest in the left lateral decubitus or supine position using standard parasternal and apical views. All the measurements were made by the leading-edge and averaged over three cardiac cycles.

## RESULTS

Out of a total of 600 patients who were included in the study, 428 (71.3%) were female and 172 (27.6%) were male. The mean age was 44.24±14.24 (range 8-83) years. The majority of the RHD patients belonged to 31-50 years (51.6%). The baseline characteristics of patients with RHD are shown below (Table 1).

**Table 1:** Baseline characteristics of patients with RHD.

Characteristics	n (%)
Male	172 (27.6)
Female	428 (71.3)
Smoker	106 (17.6)
Alcohol use	66 (11)
History of rheumatic fever	12 (2)
Characteristics	Mean±SD
Mean age in years (Total)	44.24±14.24
Male	43.33±13.74
Female	44.61±14.43
Mean ^*^BMI	21.5±4.62
Waist: hip ratio	0.85±0.08
Mean systolic blood pressure	112.6±16.1
Mean diastolic blood pressure	73.4±10.4
Mean blood pressure	86.4±11.04
Mean heart rate, ^†^BPM	87.6±18.7
Creatinine	0.9±0.15
eGFR^‡^	77.1±25.2
Mean hemoglobin, gm/dl	12.3±1.8
Mean ^**^TLC	7693±3826
Mean Platelets 234.6	234.6 × 10^3^±55.03
Mean ^††^CHA_2_DS_2_VaSC score	0.98±0.81

* BMI: body mass index;

† BPM: beat per minute;

‡ eGFR: estimated glomerular filtration rate;

** TLC: total leucocyte count;

†† CHA_2_DS_2_VaSC: chronic heart failure, age, hypertension, diabetes mellitus, stroke, vascular disease, sex category.

**Figure 1:**
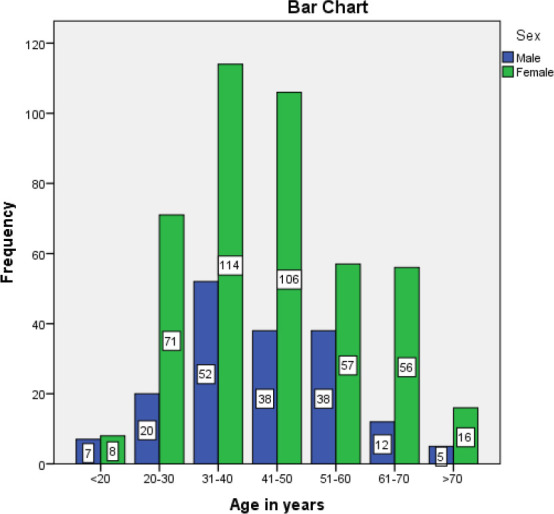
Figure 1. Distribution of RHD according to age groups.

The distribution of patients with RHD according to different age groups is shown below (Figure 1).

**Figure 2:**
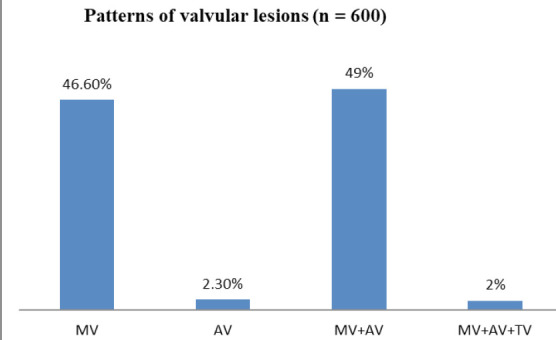
The pattern of different valves involved in patients with RHD.

Isolated mitral valve 280 (46.6%) was the most commonly affected valve in our study. Dual involvement of mitral and aortic valve was present in 294 (49%). Only 14 (2.3%) had involvement of isolated aortic valve and 12 (2%) had involvement of combined mitral, aortic, and tricuspid valves and none had pulmonary valve involvement as shown in (Figure 2).

Among patients who had either isolated or combined valvular lesions, MS was the most common abnormality 508 (84.6%) followed by MR 418 (69.6%), AR 320 (53.3%), and AS 63 (10.5%). There was overlapping of the stenotic and regurgitant lesions of different severity in a single valve or combined lesions. TR was present in the majority of patients 590 (98.3%) including both organic valvular involvement 12 (2%) and functional 578 (96.3%). Assessment of the severity of various valvular lesions showed that 247 (41.2%) patients had severe MS, 119 (19.8%) patients had severe MR, 14(2.3%) patients had severe AS and 11 (1.8%) had severe AR. The severity of valvular lesions is illustrated below in (Table 2).

**Table 2:** The severity of valvular lesions in patients with RHD.

Valve lesions	Total n (%)	Mild n (%)	Moderate n (%)	Severe n (%)
^*^MS	508 (84.6)	113 (18.8)	148 (24.6)	247 (41.2)
^†^MR	418 (69.6)	182 (30.3)	117 (19.5)	119 (19.8)
^¶^AR	320 (53.3)	246 (41)	63 (10.5)	11 (1.8)
^**^AS	63 (10.5)	18 (3)	31 (5.2)	14 (2.3)
^††^TR	590 (98.3)	-	-	-
Organic	12 (2)	-	-	12 (100)
Functional	578 (96.3)	212 (36.6)	186 (32.1)	180 (31.1)

* MS: mitral stenosis;

† MR: mitral regurgitation;

¶ AR: aortic regurgitation;

** AS: aortic stenosis;

†† TR: tricuspid regurgitation.

Overall, 150 (25%) patients had tachycardia (heart rate > 100/minute), prevalence of AF was 220 (36.6%). The majority of patients (89.6%) had enlarged left atrium (>40 mm). Reduced left ventricular systolic function was noted in 473 (78.8%), pulmonary artery hypertension in 260 (41.6%), left atrial clot in 30 (9.1%) patients.

The majority of patients 493 (82.2%) were being treated with various medical therapies. History of prior PTMC was noted in 56 (9.3%) of patients. Different Surgical procedures were performed in 51 (8.5%) of patients as shown in (Table 3). The use of anticoagulation therapy was documented in 212 (35.3%) of high-risk eligible patients. Only 174 (29%) of the study population was receiving secondary prophylaxis.

**Table 3:** Current treatment of patients with RHD.

Treatment	n (%)
Medical treatment	493 (82.2)
Beta-blockers	355 (59.2)
Digoxin	119 (19.8)
Calcium channel blockers	37 (6.2)
Anticoagulation (Warfarin)	212 (35.3)
Secondary prophylaxis	174 (29)
^*^PTMC	56 (9.3)
^†^MVR	35 (5.8)
^¶^DVR	7(1.2)
^**^CMC	6(1)
^††^AVR	3 (0.5)

* PTMC: percutaneous transluminal mitral commissurotomy;

† MVR: mitral valve replacement;

¶ DVR: double valve replacement;

** CMC: closed mitral commissurotomy;

†† AVR: aortic valve replacement.

## DISCUSSION

This is a hospital-based study providing data on clinical patterns of RHD concerning age and gender distribution, the pattern of valves involvement, and treatment practices in the eastern region of Nepal. Similar to other studies performed in Nepal, our study revealed that RHD was more common among patients of the age group between 20 to 50 years accounting for around two-thirds of total cases. A study conducted by Koirala PC, et al.^6^ in National Heart Center showed that RHD was more common among patients of age group 10-40 years with a female predominance. Similarly, studies done by Laudari S, et al.^7^ and Kafle, et al.^8^ in different regions of Nepal showed that the majority of the patients belonged to productive age of life with a common age group being in between 21 to 40 years. In our study, females were more commonly involved than males with female to male ratio of 2.4. That could be probably due to lack of access to health care and proper treatment of sore throat in female children because of gender biases or other factors that still prevails in our Nepalese community.

In our study, mitral valve was affected in the majority of patients (95.6%) followed by an aortic valve (51.3%) similar to study done in National Heart Center^6^ that showed that mitral valve was the most commonly affected valve (98.20%) followed by the aortic valve (53.90%). Overall, mitral stenosis was the predominant lesion (84.6%) followed by mitral regurgitation (69.6%). Prevalence of predominant MS was found to be more in our study than in a large retrospective analysis in south India that showed predominant MS was present in 41.5% of the population age for more than 18 years. In our study, an isolated mitral valve was the most commonly involved valve (46.60%) followed by an isolated aortic valve (2.30%) similar to other studies conducted in other parts of Nepal.^6,7^ Laudari S, et al.^7^ showed that an isolated mitral valve was the most commonly involved valve (46.80%) followed by an isolated aortic valve (9.36%). Similarly, Koirala PC, et al.^6^ revealed that the isolated mitral valve was far more (46.05%) than the isolated aortic valve (1.9%).

Concerning isolated aortic valve disease, our study showed a similar prevalence (2.3%) like the study done in south India (2.8%). Multi valvular involvement (mitral plus aortic valve) was present in 49% of patients in this study similar to a study done by Laudari, et al.^7^ in other parts of Nepal which showed that combined mitral plus aortic valve lesions were the most common. This is also similar to the study done in India where commonest valvular lesions were combined MS and MR (42.9%).^10^ Treatment decision of patients with RHD is made based on the clinical status, the severity of valve lesions, and resources available at the center providing the care. Surgical treatment is indicated if the patient is symptomatic or LV dysfunction is present. Few tertiary care centers outside the Kathmandu valley regularly perform valvotomies and valve surgeries in clinically indicated patients. The majority of patients are treated medically even if indicated for valvotomies or surgical intervention due to various reasons like financial constraints, lack of manpower or resources, limited knowledge, advanced disease at presentation with high peri-procedural complications, etc. In our study, the majority were treated with medical therapy (82.2%) with the rest had undergone some type of cardiac interventions in the past. Percutaneous transluminal mitral valvoplasty (PTMC) is performed if the valve anatomy is favorable and there is no significant MR.^11^ Around 10% of our patients had undergone PTMC with favorable anatomy before enrollment. In our study, out of 220 patients of MS with AF, only 35.3% patients were on warfarin (DOACs rarely used in our settings) at the time of enrollment as seen in the study done in the rural part of Nepal where only 22.7% patients with RHD and AF were on oral anticoagulants^12^ that underscores the marked underuse of anticoagulation in clinically indicated patients.

The previous study^13^ suggests that a history of rheumatic fever (RF) is missed in almost more than 50 percent of patients with RHD. Interestingly, only 2 percent of our patients could recall the childhood manifestations of RF suggesting that our population has a higher prevalence of subclinical carditis. Secondary prophylaxis is indicated in RHD patients who had either history of RF or documented RHD. Three weekly intramuscular injections of benzathine benzylpenicillin are indicated as the most effective therapy for secondary prevention.^1^ Only 29% of our patients were prescribed penicillin therapy that too oral penicillin due to factors like fear of anaphylaxis, poor access to health care facility every three weeks, etc.

The main limitation of the study is that this is a hospital-based cross-sectional study and provides information related to patients referred to a tertiary care hospital. Hence these results may not be generalized to the general population because of referral bias.

## CONCLUSIONS

RHD is still a major problem in Nepal. It is important to be familiar with patterns and severity of valvular lesions and treatment modalities provided to improve the care of such patients in our settings. MV was most commonly affected both in isolation and combination. The majority of patients who are eligible for cardiac interventions are treated medically with suboptimal use of anticoagulation and secondary prophylaxis. This demands to look at the reasons behind not giving treatment as par with the evidence-based guidelines and address the obstacles related to it. Improved knowledge of treatment based on the severity of disease in addition to primary and secondary prevention helps to reduce the burden of RHD.
